# A Method for Recognizing Dead Sea Bass Based on Improved YOLOv8n

**DOI:** 10.3390/s25144318

**Published:** 2025-07-10

**Authors:** Lizhen Zhang, Chong Xu, Sai Jiang, Mengxiang Zhu, Di Wu

**Affiliations:** College of Engineering Science and Technology, Shanghai Ocean University, Shanghai 201306, China; lzzhang@shou.edu.cn (L.Z.); m230851604@st.shou.edu.cn (C.X.); m230851605@st.shou.edu.cn (S.J.); m230851544@st.shou.edu.cn (M.Z.)

**Keywords:** dead sea bass, target detection, deep learning, YOLOv8

## Abstract

Deaths occur during the culture of sea bass, and if timely harvesting is not carried out, it will lead to water pollution and the continued spread of sea bass deaths. Therefore, it is necessary to promptly detect dead fish and take countermeasures. Existing object detection algorithms, when applied to the task of detecting dead sea bass, often suffer from excessive model complexity, high computational cost, and reduced accuracy in the presence of occlusion. To overcome these limitations, this study introduces YOLOv8n-Deadfish, a lightweight and high-precision detection model. First, the homemade sea bass death recognition dataset was expanded to enhance the generalization ability of the neural network. Second, the C2f-faster–EMA (efficient multi-scale attention) convolutional module was designed to replace the C2f module in the backbone network of YOLOv8n, reducing redundant calculations and memory access, thereby more effectively extracting spatial features. Then, a weighted bidirectional feature pyramid network (BiFPN) was introduced to achieve a more thorough integration of deep and shallow features. Finally, in order to compensate for the weak generalization and slow convergence of the CIoU loss function in detection tasks, the Inner-CIoU loss function was used to accelerate bounding box regression and further improve the detection performance of the model. The experimental results show that the YOLOv8n-Deadfish model has an accuracy, recall, and mean precision of 90.0%, 90.4%, and 93.6%, respectively, which is an improvement of 2.0, 1.4, and 1.3 percentage points, respectively, over the original base network YOLOv8n. The number of model parameters and GFLOPs were reduced by 23.3% and 18.5%, respectively, and the detection speed was improved from the original 304.5 FPS to 424.6 FPS. This method can provide a technical basis for the identification of dead sea bass in the process of intelligent aquaculture.

## 1. Introduction

Fish, as a key component of aquaculture, is an important source of high-quality protein supply globally and plays a vital role in ensuring food and nutritional security [1]. Currently, global fisheries are transitioning towards intensive development, integrating factory-based and intelligent technologies, and this transition marks a major change in aquaculture practices [2]. Recirculating aquaculture systems (RAS) have become an important choice for fish farmers because they can provide a stable aquaculture environment throughout the year, accurately regulate temperature, manage water quality, and improve feed utilization efficiency [3]. However, abnormal fish morphology or even mortality often occurs during the aquaculture process, which, if not detected and resolved in time, will pose serious challenges to the sustainable development of aquaculture. Therefore, real-time monitoring of fish mortality has become one of the key aspects in recirculating water aquaculture systems. This monitoring mechanism can not only effectively reduce the risk of water pollution caused by fish mortality but also prevent the occurrence of large-scale mortality events. Traditional manual observation methods have significant limitations and are not only time-consuming and labor-intensive but also face sustainability challenges as the number of personnel engaged in monotonous dead fish cleanup is decreasing [4].

In the last decade, many studies have used computer vision (CV) for automatic identification, classification, and production-status monitoring of fish due to its inherent advantages of speed, objectivity, and high accuracy [5]. Initially, computer vision technology relied on traditional machine learning techniques in target detection, which—in combination with the Internet of Things (IoT), cloud computing, optical sensors, and robotics—has been used to monitor fish population, behavior, feeding, and stress response [6]. However, traditional machine learning methods require a manual extraction of features, which not only requires some specialized knowledge from operators but also still has limited robustness and accuracy in complex aquaculture environments [7].

To overcome the limitations of traditional machine learning methods for behavior detection in computer vision, some researchers have begun to focus on the use of deep learning techniques to analyze the behavior and health of farmed fish [8]. Deep learning, especially the convolutional neural network (CNN), is capable of automatically extracting high-dimensional features from a large amount of complex image or video data, avoiding the limitations of manual feature extraction, thus effectively improving the accuracy and efficiency of dead fish detection [9]. Through deep learning models, especially with the application of convolutional neural networks, the behavior and health status of fish can be accurately analyzed, which reduces human intervention and shows stronger robustness and adaptability in complex aquaculture environments [10].

Currently, deep learning-based fish detection methods mainly use two-stage or single-stage algorithms. Common two-stage algorithms include R-FCN [11], Faster R-CNN [12], and R-CNN [13], while typical single-stage algorithms include the You Only Look Once (YOLO) series [14] and the Single-Shot MultiBox Detector (SSD) [15]. The two-stage algorithms usually separate object detection from classification by generating a series of candidate bounding boxes, i.e., the “region suggestion part”, and then feeding these regions into the CNN for detection and classification. For example, Zhao [16] proposed a grass carp feeding state classification method combining inter-frame optical flow features with an augmented recurrent neural network (RNN), which achieved a high classification accuracy in an outdoor pond aquaculture environment, with an average detection accuracy of 91% and a recall rate of 92.2%. In addition, Jager [17] used an improved R-CNN algorithm and utilized background differencing to generate candidate frames for underwater fish detection. Li [18] conducted a preliminary study on sea cucumber identification in a laboratory environment and proposed a sea cucumber identification and behavior analysis method based on Faster R-CNN, achieving an average accuracy of 99.93% (mAP@0.5). The FFA-SSD algorithm proposed by Shuapeng Yang [19] significantly improves the accuracy of dead fish detection on the surface of the water by improving the multi-scale feature fusion module, introducing the channel attention mechanism, and designing the small target data augmentation method, which reaches an accuracy of 93.5%. Although the two-stage method excels in accuracy, its high computational volume and the complexity of the proposed region extraction process limit its efficiency in practical applications.

In single-stage algorithms, CNNs are often used in regression tasks, where the target detection problem is treated as a regression problem. Such models directly output the target location through end-to-end training, thus simplifying the system architecture and improving the performance. For example, Cai [20] proposed an improved YOLOv3 fish detection model that uses MobileNet V1 as the backbone network. MobileNet re-selects feature maps by adjusting the receptive field of the image and optimizes the feature map selection strategy, while ensuring accuracy, and achieves higher detection speeds based on the ImageNet dataset. Li [21] used Swin Transformer as the backbone network of YOLOv5 and improved the structure of the PAN (Path Aggregation Network) in order to enhance the fusion ability of multi-scale features, which improves the feature extraction effect of underwater targets. The improved scheme achieves an mAP of 87.2% in the recognition of targets such as sea cucumbers, sea urchins, scallops, and starfish, which is a 2.3% improvement over the previous model. For dead fish detection, Zhao [22] proposed a lightweight YOLOv4 target detection model based on deformable convolution specifically for dead sea bass recognition; although the method outperforms the original YOLOv4 in terms of detection speed, there are still some shortcomings in detection accuracy and feature extraction capability.

In recent years, for the underwater fish detection task, a number of studies have been optimized based on the YOLOv8n model. Zhang [23] proposed YOLOv8n-MEMAGD, which improves the localization ability in fish aggregation scenarios by introducing the GELU activation function, the attention modules (C2f-FAM and C2f-MSCA), and the MPDIoU loss function. However, their method mainly focuses on fish counting and still lacks the ability to recognize fine-grained differences in target occlusion and dead fish. Chen [24] developed Light-YOLO, which improves the detection accuracy of fishing nets in complex underwater environments through sparse connectivity and the CoTAttention and SEAM attention modules. However, the model does not specifically consider the multi-scale fusion problem in fish detection and lacks a mechanism to handle small and overlapping targets. The BSSFISH-YOLOv8 model proposed by Zhang Zehao et al. [25] enhances small fish targets in low-light and blurred environments, and its structure introduces a sparse attention and small target detection layer (STDL) to achieve enhanced perception of small targets. However, their approach still relies on a more complex attention mechanism with high training cost, and lacks systematic optimization for realistic aquaculture scenario problems such as occlusion and background interference. Shah et al. [26] designed YOLOv8-TF, which introduces a Transformer module and a class-awareness loss function to effectively solve the class imbalance problem in fish species recognition. However, the model has a heavy structure and a large number of parameters, which are not suitable for resource-constrained edge deployment with real-time demanding aquaculture monitoring systems.

Although target detection techniques have made significant progress in several fields, including breakthroughs in accuracy, there are still many shortcomings in the application of aquaculture image recognition tasks. On the one hand, the existing methods generally suffer from high model complexity, lack of targeted optimization for typical aquaculture scenarios such as occlusion, small targets, background noise, etc., and lack of specific modeling mechanisms—especially in the identification of dead fish; on the other hand, the current research often does not fully consider the complex environmental factors in the experimental design such as fish occlusion, water turbidity, and light changes, and these visual interferences are highly prevalent in recycled water. Additionally, these visual disturbances are highly prevalent in recirculating water aquaculture systems, which severely limit the accuracy and robustness of the detection models. At the same time, in order to meet the needs of mobile deployment, it is necessary to solve problems such as slow real-time detection, high model complexity, and a large number of parameters. This paper proposes an improved YOLOv8n neural network model, YOLOv8n-Deadfish, which validates its dead fish recognition performance in experimental scenarios that simulate real aquaculture conditions with the aim of providing a methodological basis and model reference for future deployment in actual aquaculture systems. The YOLO framework, as a widely used single-stage detection method, has the advantages of speed and simplicity. However, its operating parameters and computing resources are still demanding, making it difficult to deploy on embedded devices and difficult to meet the real-time detection requirements of industrial environments. To address this problem, the YOLOv8n-Deadfish neural network model proposed in this paper significantly improves detection speed and reduces computational complexity through its lightweight design, while maintaining high detection accuracy, achieving good detection accuracy, high processing efficiency, and low computational cost.

## 2. Improved YOLOv8n Algorithmic Modeling

### 2.1. Introduction of YOLOv8n Model

YOLOv8 is a target detection framework proposed by Ultralytics in early 2023. The model has made several innovations and improvements based on the inheritance of the advantages of the YOLO series of models, and it is one of the most popular target detection algorithms. Its network structure mainly consists of four parts: input layer, backbone network, neck, and detection head [27].

After the image passes through the input layer, it is first passed through the backbone network for feature extraction to generate feature maps. Then, these feature maps are passed to the neck network, which is responsible for integrating feature information from different layers and further passing the fused features to the detection head. The detection head utilizes this feature information for target identification and position prediction. YOLOv8 is divided into YOLOv8n, YOLOv8s, YOLOv8m, YOLOv8l, and YOLOv8x according to the model size. The depth and width of these five models are increased in turn, and the detection accuracy is gradually improved but the time spent on training is also increased in turn. According to the actual needs of agricultural production, the YOLOv8n model is chosen as the base model because of its good detection accuracy and faster recognition speed, which is easy to deploy on mobile devices for automatic detection.

### 2.2. YOLOv8n-Deadfish Network Model

**Figure 1:**
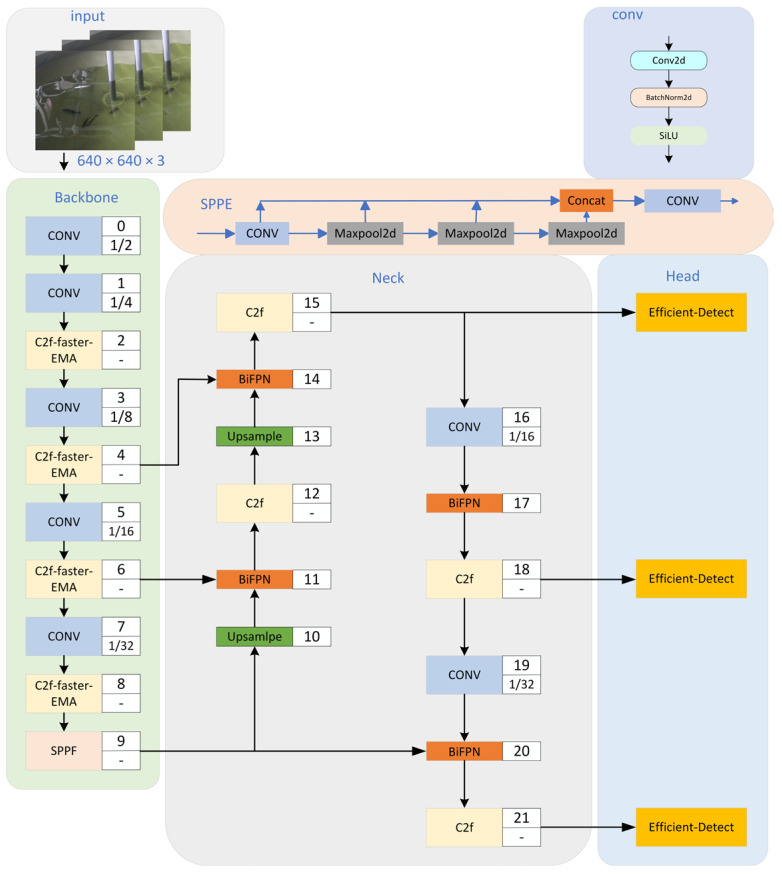
Structure of YOLOv8n-Deadfish network.

In recirculating water aquaculture, dead sea bass image capture often faces the problem of cluttered backgrounds and other interferences, and in such environments, CNNs may introduce redundant features and noise when extracting image features, which poses a challenge in accurately recognizing dead fish bodies. Therefore, this study chooses to design and improve the model for YOLOv8n, which is mainly improved and optimized in the following aspects in order to achieve better detection performance and lower computational cost. Aiming at the problems of the large number of parameters and high computational complexity of ordinary convolutional neural networks, a lightweight scheme of staged optimization is proposed. Firstly, the standard Bottleneck in the C2f module is replaced by the more efficient FasterNet Block module through the structural reconfiguration strategy, thus constructing a lightweight C2f-faster base module that effectively compresses the model scale. In order to further strengthen the feature extraction capability, the EMA attention mechanism is innovatively integrated into the forward propagation process of the C2f-faster module to form a C2f-faster–EMA composite module with attention enhancement characteristics. This step-by-step optimization strategy significantly improves the feature expression ability of the network through the attention-guided feature enhancement mechanism while maintaining the lightweight property of the model. Next, a weighted bidirectional feature pyramid network (BiFPN) is introduced to realize the full fusion of deep and shallow features, ensuring that the network more effectively handles the complex information in the images of dead sea bass and enhances the effect of feature fusion. Secondly, in order to improve the detection accuracy of the model and accelerate the speed of bounding box regression, Inner-CIoU (inner for complete intersection over union) loss function is introduced to make up for the problems of weak generalization and slow convergence speed of CIoU (complete intersection over union) loss function in the detection task. In this paper, we optimize the YOLOv8n network and construct a dead sea bass detection model named YOLOv8n-Deadfish, as shown in Figure 1.

### 2.3. Improvement in the C2f Module

In YOLOv8n, the main role of the C2f structure is to fuse feature information from different channels in order to extract image features more efficiently. The C2f module consists of two convolutional layers and a number of Bottleneck modules, where the Bottleneck modules act as a residual block, which helps to increase the depth and complexity of the network. The module extracts and fuses the information in the input feature maps through the iteration of convolutional operations and Bottleneck modules to generate richer feature maps. However, in the dead sea bass image, the model faces significant noise interference due to the complexity of the background and the occlusion between the targets, resulting in a lower detection accuracy of the dead sea bass. In addition, the introduction of the C2f module increases the number of parameters and computational complexity of the model. Therefore, the strategy to optimize the C2f feature extraction module focuses on the lightweight and fine-grained feature extraction of the module.

#### 2.3.1. C2f-Faster Module Lightweighting

**Figure 2:**
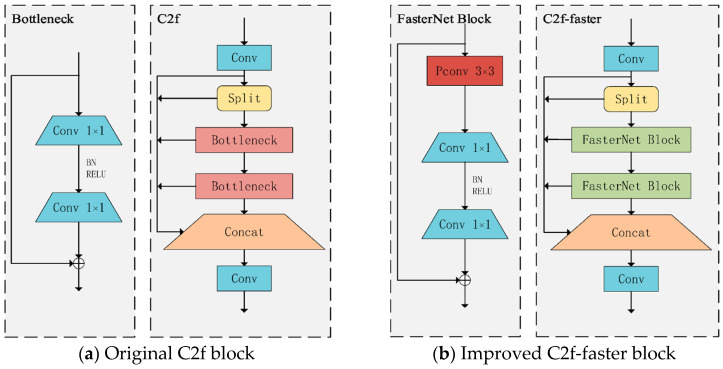
Original C2f and improved C2f-faster block.

In order to improve the computing speed of the neural network, the C2f-faster module is proposed, which effectively reduces the number of floating-point operations and parameter counts, thus reducing the complexity of the overall model. Replacing the Bottleneck module in C2f with the FasterNet Block in FasterNet [28] achieves an effective reduction in model size. Figure 2 illustrates the original C2f model and the corresponding model with the improved C2f-faster module.

**Figure 3:**
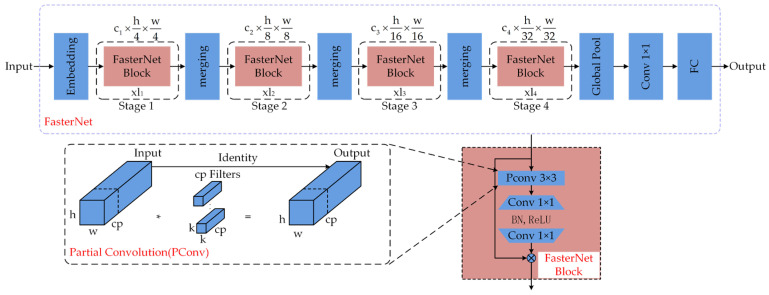
Working principle of PConv and FasterNet network structure.

FasterNet is a new neural network proposed at CVPR 2023. It runs faster and more stably on multiple devices—such as GPUs, CPUs, and ARM processors—than other networks such as EfficientVit [29]. It mainly uses partial convolution (PConv). Spatial features are more effectively extracted by reducing redundant calculations and simultaneous memory access. Its working principle is shown in Figure 3.

PConv performs regular convolution on only some of the input channels to extract spatial features, leaving the remaining channels unchanged. The first or last consecutive c_p_ channel is usually chosen as the feature map representative when computing continuous or periodic memory accesses. Therefore, the FasterNet Block, with the introduction of PConv, not only improves the computational efficiency but also plays a key role in spatial feature extraction. The number of floating-point operations (FLOPs) and memory accesses of PConv are as follows, and the FLOPs of PConv are shown in Equation (1):

(1) $$FLOPs=h\times w\times k^{2}\times c_{p}^{2}$$

where h and w represent the width and height of the feature map, respectively; k is the kernel size; and cp is the number of channels in the convolution operation. In practical implementation, r = cp/c = 1/4. Therefore, the FLOPs of PConv are only 1/16 of that of a regular convolution.

The memory access of PConv is shown in Equation (2), as outlined below:

(2) $$h\times w\times 2c_{p}+k^{2}\times c_{p}^{2}\approx h\times w\times 2c_{p}$$

where h and w denote the width and height of the feature map, respectively; k is the convolution kernel size; and c is the number of channels for the convolution operation. The number of memory accesses of PConv is only 1/4 of that of the conventional convolution, and the rest of the c–c channels are not involved in the computation; thus, there is no need to perform memory accesses. It can be seen that the introduction of FasterNet Block with PConv in the feature extraction network can significantly reduce the amount of computation and memory access, thus making the original model network more lightweight.

#### 2.3.2. EMA (Efficient Multi-Scale Attention) Feature Extraction Module

**Figure 4:**
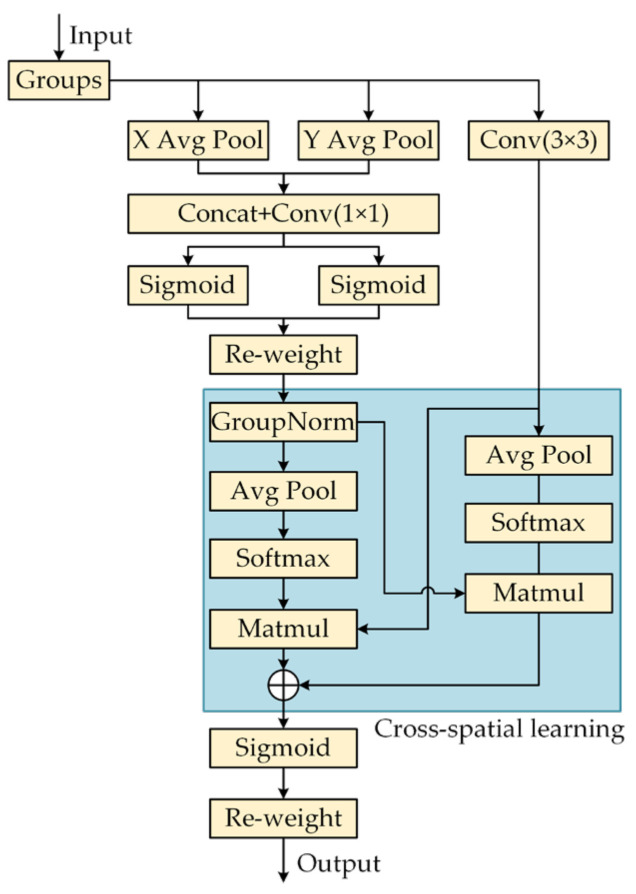
EMA network architecture.

In order to enhance the correlation between pixels, researchers have introduced attention mechanisms (e.g., CBAM [30] and SE [31]) into convolutional neural networks, which show significant advantages in the fusion of cross-dimensional attentional weights with input features. However, these attention modules lead to a significant increase in computational requirements due to the inclusion of a large number of pooling operations. To address the problem of high computational cost, researchers proposed the EMA feature extraction module, whose network architecture is shown in Figure 4 [32]. The EMA employs three parallel paths for extracting the attention channel weights, which are used for grouping the feature maps. Among these paths, the first two are 1 × 1 branches and the third is a 3 × 3 branch. In the 1 × 1 branch, two 1D global average pooling operations are used to encode the channel along both spatial directions. For the 3 × 3 branch, it utilizes only one stacked 3 × 3 kernel to capture the multi-scale feature representation. In addition, EMA incorporates cross-space learning to aggregate cross-space information from different spatial dimensions for more comprehensive feature aggregation.

The original C2f feature extraction module extracts features from the input image, which makes the network’s ability to extract small pixel information about the death of the sea bass slightly insufficient. In order to enable the algorithm to capture pixel information in multiple dimensions, and to solve the problem that dead sea bass images are susceptible to cluttered background interference and features are difficult to be extracted, this paper adds the EMA attention module to the forward propagation process of C2f-faster, constituting the C2f-faster–EMA lightweight feature extraction module. The multi-level feature pyramid structure fully exploits the correlation information of visual representations in spatial and channel dimensions, effectively solves the feature confusion problem due to complex background interference in underwater sea bass death detection, and strengthens the texture and contour representations of small-scale targets through dynamic feature enhancement mechanism, which achieves a significant improvement in detection accuracy and robustness in real acquisition scenarios.

### 2.4. Improved Multi-Scale Feature Fusion Approach

**Figure 5:**
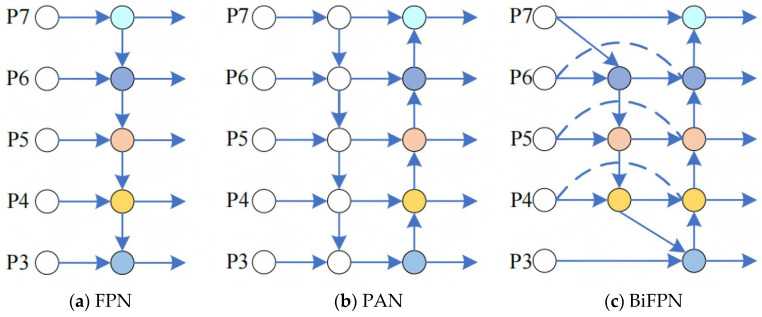
Comparison of three feature pyramid structures.

In the dataset for dead sea bass recognition, there are a large number of small target samples in the foreground that occupy a small area in the overall image and carry limited feature information. After multiple layers of convolution, downsampling, and pooling operations, some information may be lost. Multi-scale feature fusion technology can integrate sea bass information from different levels, enabling the network to simultaneously utilize shallow dead fish location information and deep dead fish features, thereby effectively mitigating the problem of information loss. As shown in Figure 5a,b, YOLOv8n implements multi-scale feature fusion through the feature pyramid network (FPN) and Path Aggregation Network (PAN) but its performance is still insufficient in the dead sea bass recognition task. To this end, this paper introduces BiFPN to optimize the feature fusion mechanism of YOLOv8n. Its structure is shown in Figure 5c, which is designed to improve the accuracy of dead sea bass detection.

The original FAN structure is followed by top-down feature fusion, followed by bottom-up fusion to form a bidirectional feature fusion, and PAN can be regarded as an extension of this bidirectional FPN. For multi-scale feature fusion, BiFPN adopts a more refined strategy—not simply adding or splicing features—by weighting multi-scale input features and eliminating single-input nodes, which contribute less to the network because they are not involved in feature fusion. In addition, when the input and output nodes are at the same scale, BiFPN introduces additional paths to optimize feature fusion while avoiding adding too much computational cost. Finally, BiFPN further improves the feature fusion by repeating the feature network with bidirectional paths multiple times.

### 2.5. Improvement in the Loss Function

Before the improvement, the YOLOv8n network predicts the anchor box coordinate loss using the CIoU (complete intersection over union) loss [33], which is calculated as shown in Equations (3)–(6):

(3) $$\alpha =\frac{v}{(1-IoU)+v}$$

(4) $$v=\frac{4}{\pi^{2}}{\left(\arctan\frac{w^{gt}}{h^{gt}}-\arctan\frac{w}{h}\right)}^{2}$$

(5) $$CIoU=IoU-\frac{\rho^{2}(b,b^{gt})}{C^{2}}-\alpha v$$

(6) $$CIoU=IoU-\frac{\rho^{2}(b,b^{gt})}{C^{2}}-\alpha v$$

where *α* is the weight function; v is a parameter used to measure the consistency of the aspect ratio; *b* and *b^gt^* represent the centroids of the predicted and real frames, respectively; *ρ* stands for calculating the Euclidean distance between the two centroids; and *C* stands for the diagonal distance of the smallest closure region that can contain both the predicted and real frames.

The CIoU loss function takes into account the overlap area, centroid distance, and aspect ratio. However, when the aspect ratio of the predicted frame to the real frame is linear, its penalty term may degenerate to 0. In addition, the regression loss is negatively affected regardless of the quality of the anchored frame [34]. In order to solve these problems, Zhang [35] proposed the Inner-IOU loss function, which is based on the concept of the auxiliary bounding box and generates the auxiliary bounding box by introducing a scaling factor that is able to effectively reflect the quality of the regression results of the real bounding box. The computation of the Inner-CIoU loss function is shown in Equation (7):

(7) $$L_{Inner-CIoU}=L_{CIoU}+(IoU-IoU^{Inner})$$

where *L_CIoU_* is the *CIoU* loss; *IoU* is the ratio of the area where the predicted box intersects the ground truth box to the area where they are merged; and is the ratio of the area where the predicted box intersects the ground truth box to the area where they are merged obtained by the scaling factor. Optimizing CIoU by using an Inner-CIoU with a scaling factor less than 1 can accelerate sample regression, improve the generalization ability of the network, and accelerate the convergence of the model. Applying this optimization scheme to sea bass death target detection can help improve the performance of the target detection model in different scenes and scales and improve the robustness of the model.

It is worth emphasizing that the Inner-CIoU loss function employed in this study is differentiable with respect to the predicted bounding box coordinates. Although IoU-based loss functions involve geometric computations such as intersection and union areas, modern formulations like CIoU and Inner-CIoU are constructed using continuous functions—such as the center coordinates, widths, heights, and overlapping areas of the bounding boxes—which are differentiable almost everywhere. In practical implementation, the derivatives of these quantities with respect to the predicted coordinates are well-defined and were successfully incorporated into mainstream deep learning frameworks.

Since the predicted bounding box coordinates are direct outputs of the neural network and are functions of the network parameters (i.e., weights), the chain rule in backpropagation ensures that gradients from the loss function can be effectively propagated back through the bounding box predictions to the network parameters. As a result, the Inner-CIoU loss function is fully compatible with gradient-based optimization algorithms such as SGD and Adam, allowing for efficient end-to-end training of the detection model.

## 3. Experimental Results and Analysis

### 3.1. Experimental Environment and Parameter Settings

The training platform configuration includes an i5-12400F CPU, NVIDIA RTX 4060 GPU (8 GB graphics memory) running on a Windows 10, 64-bit system with Python 3.8, PyTorch 1.13 framework, and CUDA version 11.7. The training parameter settings are shown in Table 1.

### 3.2. Dataset Preparation

**Figure 6:**
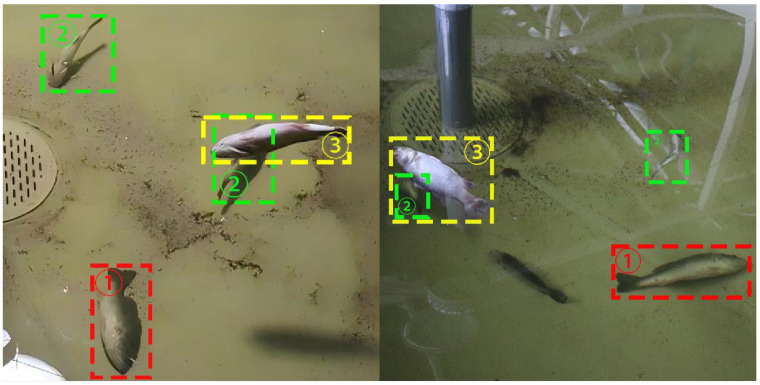
Dead sea bass positions (the dead sea bass positions 1, 2, and 3 in the figure are labeled dead_sideways, dead_belly_up, and dead_float, respectively).

The experiments were conducted at the Ecological Aquaculture Center in Puyue Town, Chongming District, Shanghai. During data acquisition, deliberate efforts were made to approximate real-world aquaculture conditions by adjusting camera angles, lighting intensity, and water background complexity, thereby enhancing the representativeness and diversity of the dataset. The data were captured using industrial camera equipment and saved in two formats: JPG and MP4. During the data collection process, factors such as fish overlap, occlusion, and lighting changes were taken into account. The Python program was used to extract frames from the MP4 format data and process them into multiple JPG format images, ultimately obtaining a homemade dataset of dead sea bass. Each image contains multiple states of the sea bass, for a total of 1565 data images. In order to improve the learning ability of the model and make it more applicable in practice, care was taken when collecting the dataset to ensure that the dead sea bass were in a variety of poses and that they were complete. As shown in Figure 6, the states of the dead sea bass include dead_sideways, dead_belly_up, and dead_float.

**Figure 7:**
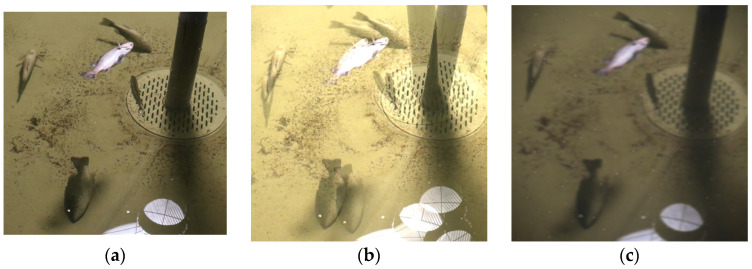
Example images generated using the EDVA strategy. (**a**) Original image. (**b**) Simulated fish occlusion and overlap. (**c**) Simulated water turbidity and bubble interference.

In order to enable the model to better adapt to complex visual disturbances in real farming environments, improve the model’s generalization ability and robustness, and avoid overfitting due to the limited number of training images, this paper designs and implements a set of environmentally disturbed visual augmentation (EDVA) strategies based on the original images. This strategy combines standard augmentation techniques—such as noise injection, random rotation, image flipping, blurring, scaling, cropping, and brightness adjustment—with domain-specific operations that simulate typical visual disturbances in aquaculture. Specifically, fish overlap and occlusion were modeled through image duplication, scaling, and overlaying (Figure 7b), while water turbidity, motion blur, and surface bubbles were simulated using a combination of Gaussian blur, motion blur, and particle overlays (Figure 7c). A total of 8160 augmented images were generated, substantially enhancing the visual diversity and complexity of the training data and supporting the evaluation of the model’s adaptability and robustness under aquaculture-relevant visual conditions.

The three dead sea bass poses, dead_sideways, dead_belly_up, and dead_float, were set to 0, 1, and 2, respectively, using the labeling tool. The dataset was labeled to generate the YOLO data format and saved as a “.txt” file. The training set, validation set, and test set are randomly divided with a ratio distribution of 8:1:1.

### 3.3. Evaluation Metrics

This paper uses evaluation metrics commonly used for object detection. Precision (P), recall (R), frames per second (FPS), mean average precision (mAP), model parameters (Parameter), and floating-point operations (FLOPs) are used as evaluation metrics to evaluate model performance. The specific calculations are shown in Equations (8)–(12):

(8) $$P=\frac{TP}{TP+FP}$$

(9) $$R=\frac{TP}{TP+FN}$$

(10) $$FPS=\frac{N}{T}$$

(11) $$AP=\int_{0}^{1}P(r)dr$$

(12) $$mAP=\frac{1}{n}\sum_{i=1}^{n}AP_{i}$$

where *TP* (true cases) represents the number of samples that the model correctly predicted as positive categories for samples that were actually positive categories; *FP* (false positive cases) represents the number of samples that the model incorrectly predicted as positive for samples that were actually negative categories; *FN* (negative cases) represents the number of samples that the model correctly predicted as negative for samples that were actually negative categories; *N* (number) represents the number of samples tested; *T* (time) represents the time required to test the full range of samples; r represents the number of thresholds; and n represents the number of categories.

### 3.4. Results Based on the YOLOv8n-Deadfish Dead Sea Bass Detection Model

**Figure 8:**
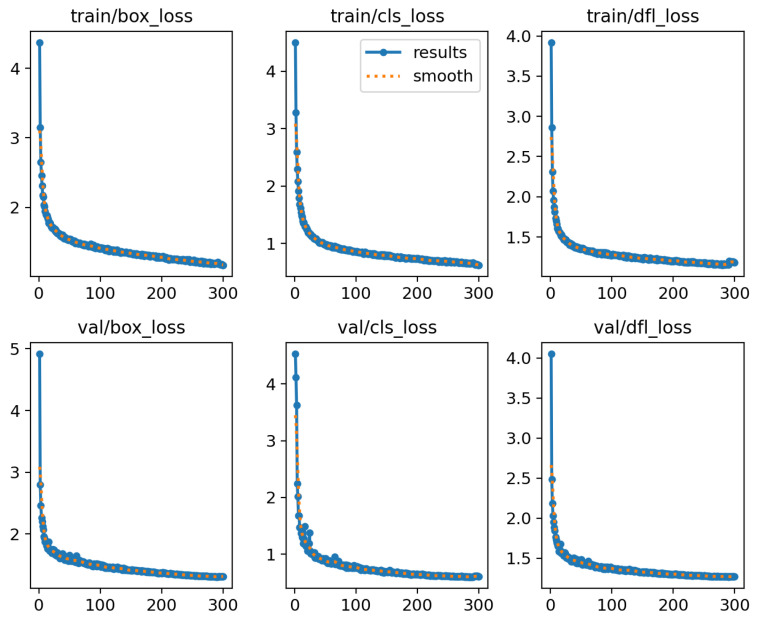
Loss variation curve during model training.

The dead sea bass posture dataset collected in this study has a high complexity, and so the overfitting problem becomes a major concern during the training process. In order to test whether overfitting occurs, loss curves were plotted during the training and validation process, and the specific results are shown in Figure 8. In the early stage of training, the loss curves show a significant decrease within the first 30 rounds, and with the training iterations, the three training losses gradually decrease and level off after 260 epochs. This indicates that the improved model does not show the overfitting phenomenon during the training process and exhibits better convergence.

**Figure 9:**
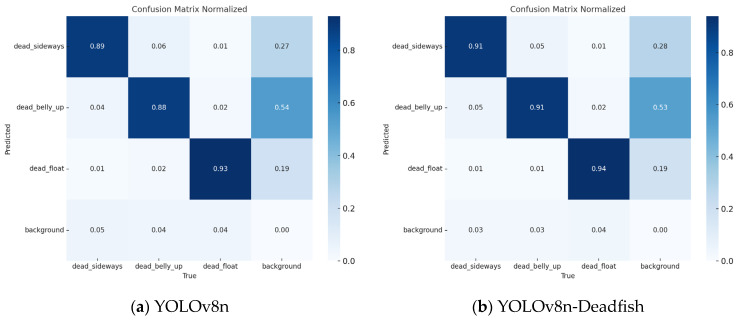
Comparison of YOLOv8n and YOLOv8n-Deadfish confusion matrices.

In order to more intuitively evaluate the practical effectiveness of the YOLOv8n-Deadfish dead sea bass detection model in the identification of each category, the confusion matrix between the YOLOv8n benchmark model and the proposed YOLOv8n-Deadfish model is compared, see Figure 9. It describes the accuracy of detecting dead sea bass in the three postures in the dataset. In the confusion matrix, the rows represent the predicted labels of the model for each category, the columns represent the true labels of the labels for each category, and the values on the diagonal represent the correct detection rate. Darker colors indicate higher rates, and lighter colors indicate lower rates. This visualization facilitates the distinction between instances belonging to each category. It can be seen that the improved model has a relatively high total number of values on the diagonal and reduces false detections due to the similarity of dead_belly_up to the background features, while improving the accuracy of dead sea bass detection.

**Figure 10:**
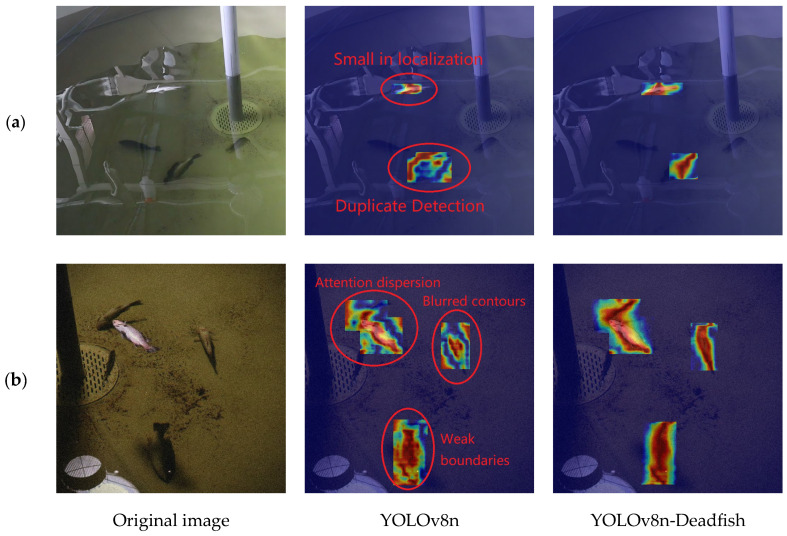
Comparison of YOLOv8n and YOLOv8-Deadfish heatmap visualization results: the colors represent a scalar quantity on an order-of-magnitude scale, with hot tones (e.g., red or yellow) indicating high activity or important regions, and other tones representing low activity or unimportant regions. (**a**) The detection image features strong water reflections, pipeline interference, and blurred fish boundaries. (**b**) The detection image has low contrast between the fish body and the background, with low overall brightness and high noise levels.

In order to evaluate the robustness of the proposed model in complex environments, two representative images of complex scenes are selected for heatmap visualization in this study. As shown in Figure 10a, the strong reflections in the water body, the interference of the pipeline structure, and the blurred boundaries of the fish body pose challenges to the detection ability of the model; in Figure 10b, the overall low brightness and high noise level of the image due to the low contrast between the fish body and the background similarly increase the difficulty of target recognition.

Figure 10 shows the comparison of thermogram visualization results between YOLOv8n and YOLOv8n-Deadfish models in complex aquaculture environments. In Figure 10a, the YOLOv8n model is interfered with by the strong reflection on the water surface when recognizing the dead sea bass in the upper left, which leads to a small detection frame and unstable localization; for the fish with occlusion and overlapping at the bottom of the image, due to the blurred edges of the dead seabass, the activation area of the model shows scattered and discontinuous features, accompanied by obvious repeated detection, which makes it difficult to achieve an effective focus on the real target. In comparison, the YOLOv8n-Deadfish model is able to focus the attention area more accurately on the location of the dead sea bass body, with clear edges and accurate localization of the heatmap, and the key areas show a stronger red response, demonstrating its superior feature extraction capability and anti-interference performance.

In the low-brightness, high-noise image in Figure 10b, although YOLOv8n generates activation responses at multiple fish locations, the activation regions have fuzzy contours and loose boundaries, and the model is not sufficiently focused on the target, making it difficult to form complete feature coverage, reflecting its limited focusing ability under complex backgrounds. On the other hand, YOLOv8n-Deadfish generates high-intensity activation regions with clear boundaries and complete structures on all four dead bass targets, and the color of the heatmap is concentrated in the red region, which shows higher focusing accuracy and feature expression power; additionally, even in the area with serious noise interference below, the model still maintains a stable response and accurately identifies the target.

In summary, the YOLOv8n-Deadfish model, by introducing improved feature extraction structure and multi-scale attention mechanism, shows stronger target focusing ability and background suppression ability when facing multiple interference factors such as strong reflections, target occlusion, complex backgrounds, or significant noise, which significantly improves the accuracy and robustness of dead sea bass detection.

### 3.5. Ablation Experiments

**Figure 11:**
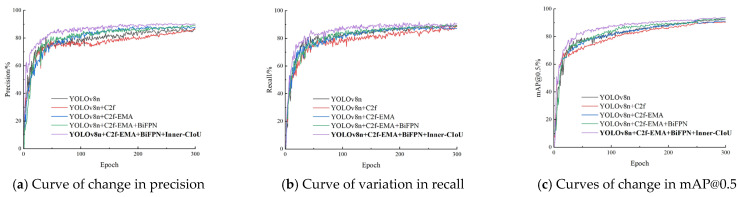
Experimental results of different improved methods on dead sea bass dataset.

In order to evaluate the effect of the improved algorithm, five ablation experiments were designed. These experiments were used to compare the effectiveness of various improvement strategies on model performance. The tests were performed using the same equipment and dataset, and other hyperparameters were kept at the same default values to ensure the comparability of the results. The results of the experiments are shown in Table 2, and the trend curves of precision, recall, and mAP@0.5 with the training cycle are plotted in Figure 11.

The experimental results show that after replacing the original C2f module of YOLOv8n with the C2f-faster module, the model parameters and floating-point operations are reduced by 26.7% and 22.2%, respectively, and the detection speed is significantly improved by 89.7 FPS. However, the accuracy indicators, precision, recall, and mAP@0.5 of the model, decreased by 1.6%, 2.2%, and 2.0%, respectively. The EMA attention mechanism was then introduced, which enhanced the model’s ability to capture features at different scales. Although the number of parameters and the amount of floating-point operations increased slightly, the detection accuracy rebounded to 91.1%, and the detection speed decreased slightly to 385.2 FPS. The further integration of the weighted bidirectional feature pyramid network (BiFPN) strengthened the bidirectional fusion of deep and shallow features, significantly improving the feature extraction ability, resulting in an increase of 1.9%, 0.5%, and 1.5% in precision, recall, and mAP@0.5, respectively, with only a slight decrease of 0.7 FPS in the detection speed. In addition, the Inner-CIoU loss function is used to further optimize bounding box regression, which effectively improves the convergence speed and accuracy of the model, ultimately achieving an mAP of 93.6% and an increase in detection speed to 424.6 FPS.

According to the trend curves of precision, recall, and mAP@0.5 with the training cycle in the figure, it can be seen that the proposed YOLOv8n-Deadfish performs best in the dead sea bass object detection task. In the early stages of training, the precision, recall, and mAP@0.5 metrics of the improved model all increased rapidly and were consistently higher than those of the original YOLOv8n and other improved methods. As the training cycle increased, each metric continued to show a significant advantage, with smoother curves and a tendency to stabilize, indicating that the model had stronger convergence performance and generalization ability.

Overall, the proposed network structure optimization strategy effectively takes into account the detection accuracy and speed, and is more suitable for the real-time accurate detection of dead sea bass targets.

### 3.6. Comparison of Different Network Models

**Figure 12:**
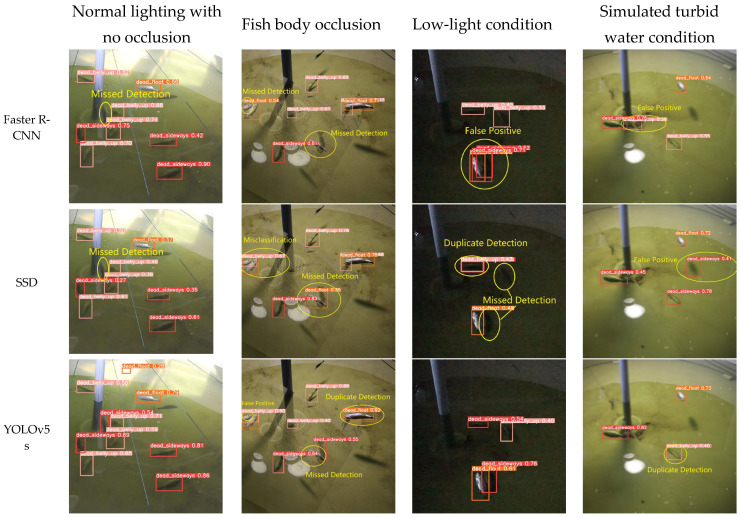

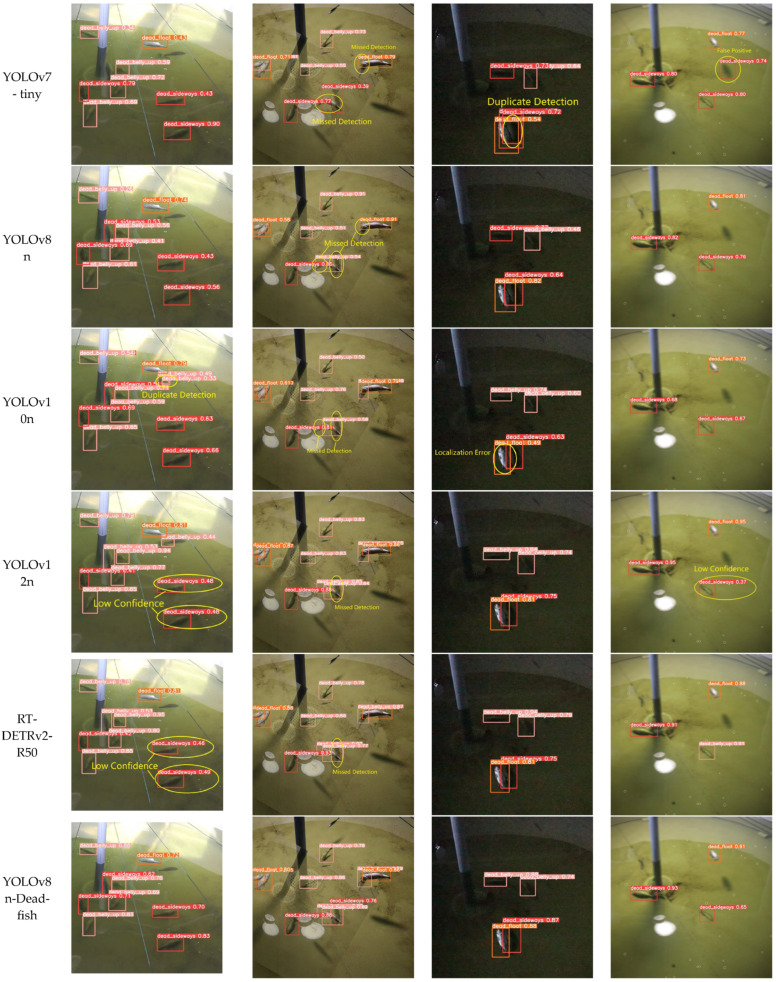
Detection effect of different mainstream models.

Table 3 and Figure 12 show the training results of the proposed YOLOv8n-Deadfish model with Faster R-CNN, SSD, RT-DETRv2-R50 [36], and other YOLO (v5–v12) series models on the self-constructed dead sea bass dataset. The comparative analysis of the performance metrics shows that the YOLOv8n-Deadfish model outperforms the other models, displaying a superior detection capability. Specifically, the model proposed in this study attained a precision (P) of 90.0%, a recall (R) of 90.4%, and a mean average precision at IoU 0.5 (mAP@0.5) of 93.6%. These figures markedly exceed those of the classical Faster R-CNN and SSD detectors and also outperform models from the same YOLO family, including YOLOv5s (88.1%, 88.4%, and 91.0%), YOLOv7-tiny (86.2%, 87.1%, and 91.8%) [37], YOLOv10n [38] (87.4%, 88.0%, and 91.0%), and YOLOv12n [39] (88.1%, 88.4%, and 91.6%); compared to the latest RT-DETRv2-R50 non-lightweight model, YOLOv8n-Deadfish is better in terms of detection precision, recall, and average precision, which are slightly higher than the RT-DETRv2-R50 model by 0.5, 1.6, and 0.9 percentage points, respectively. This indicates that the YOLOv8n-Deadfish model is able to capture the feature information of the target more effectively through the optimization of the network structure and the loss function, reducing the phenomenon of missed detection and false detection, thus achieving a higher detection precision.

While maintaining the advantage of detection accuracy, YOLOv8n-Deadfish also demonstrates significant lightweight characteristics in terms of model complexity and computational resource overhead. The model contains only 2.3 M parameters and 6.6 G of floating-point operations, which is much lower than the mainstream detectors such as Faster R-CNN (136.7 M, 401.7 G), SSD (26.3 M, 282.0 G), and RT-DETRv2-R50 (36 M, 100 G); and the number of parameters and the amount of computation are 11.5% and 1.0% lower than that of YOLOv12n and YOLOv8n, respectively. For YOLOv12n, the number of parameters and computation volume are also 11.5% and 1.5% lower than those of the latest YOLO series models, respectively. In particular, compared with RT-DETRv2-R50, YOLOv8n-Deadfish has about 94% fewer parameters and 93% less computation, which significantly reduces the model volume and computation burden with similar detection accuracy.

This lightweight advantage makes YOLOv8n-Deadfish more flexible for deployment in practical applications, especially for embedded devices, edge computing platforms, or resource-constrained systems, such as real-time video monitoring in aquaculture farms, underwater cameras, and other typical scenarios. In terms of detection speed, the model reaches 424.6 FPS, which is significantly better than all the compared models, fully demonstrating its advantages in real-time and efficiency.

In contrast, RT-DETRv2-R50 performs well in terms of detection accuracy but its large model size and high computational complexity limit its practical deployment in scenarios such as agriculture. Since the model usually relies on high-computing-power GPUs or servers, it is more suitable for environments with sufficient resources, such as urban traffic monitoring, industrial quality inspection, cloud image analysis, or medical diagnosis. In typical edge computing scenarios, such as agriculture and aquaculture, the detection model is often deployed on power-sensitive and arithmetic-limited end devices. Under such conditions, RT-DETRv2-R50 is not suitable for the above applications due to its high computational resource requirement, which not only significantly increases the deployment cost but also may affect the real-time quality and stability of the system due to high resource utilization.

To summarize, YOLOv8n-Deadfish, with its higher detection speed, lower model complexity, and good hardware adaptability, shows stronger practicality and promotion potential for practical deployment.

From the detection results of each mainstream target detection model on the dead sea bass dataset in Figure 12, it can be seen that there are significant differences in the performance of different models in complex underwater environments. Faster R-CNN and SSD show obvious limitations under disturbing conditions such as insufficient illumination, turbid water, or occlusion of the fish body, and the common problems include omission of detection, misdetection, duplicate detection, and incorrect recognition of the categories. The localization of detection frames was generally inaccurate, some fish were not recognized or misclassified, the overall confidence level was low, and the stability of detection results was poor.

In contrast, the YOLO series of models (e.g., YOLOv5s, YOLOv7-tiny, YOLOv8n, YOLOv10n, and YOLOv12n) showed improved detection performance, as evidenced by higher localization accuracy and recall. However, the models still have different degrees of problems: YOLOv5s is prone to misdetection, repeated detection, and omission in water interference or fish occlusion scenarios, and the target bounding box is not precise enough; YOLOv7-tiny has decreased detection accuracy in low-light conditions, and is prone to repeat detection of the same dead sea bass and misidentify the background region as the target; YOLOv8n has improved detection performance in the case of fish occluding each other (e.g., the second column of images). YOLOv8n, in the case of fish obscuring each other (e.g., in the second column of images), the coverage of the blurred region of the boundary is not stable enough, and there is the phenomenon of missed detection, and the overall confidence level decreases; YOLOv10n, although it is able to detect most of the targets, there is still the problem of overlapping or missed detection of the individual targets due to the offset of the bounding box; and YOLOv12n’s detection confidence is on the low side. In the first column of images, the confidence levels for multiple “dead_sideways” targets are only 0.48 and 0.41, indicating that its discriminative power is insufficient in strong reflections or complex background regions; in the second column of images, the model misses the detection of a dead sea bass individual near the pipe at the bottom of the pool, indicating that its target perception ability is limited in weakly textured or partially occluded scenes. In the second column, the model misses the dead sea bass near the pipe at the bottom of the pool, indicating that it has limited target perception ability in weakly textured or partially occluded scenes; in the fourth column, the confidence level of small-sized targets in the distance is only 0.37, which reflects that it has poor recognition stability in low-contrast conditions.

In addition, although RT-DETRv2-R50, as a newer detection framework, guarantees the target localization accuracy, its confidence level for some “dead_sideways” targets in multi-target scenes is still low (e.g., 0.46 and 0.49), which reveals its discriminative power under strong background interference. This reveals the problem of its discriminative power under strong background interference. In the second column of images, the model also has a significant omission, failing to recognize a fish body near the edge of the bottom structure of the pool, which indicates that there is still a blind spot in the complex structural region.

Overall, YOLOv8n-Deadfish performs well on several evaluation dimensions. It significantly outperforms other models in terms of target classification confidence, bounding box localization accuracy, and robustness in complex scenes. This performance improvement is mainly due to the optimized design of the network structure, including the introduction of the EMA attention mechanism to enhance the multi-scale attention capability, the adoption of the BiFPN structure to strengthen the feature fusion, the use of the C2f-faster module to improve the handling of redundant information, and the Inner-CIoU loss function to improve the bounding box regression accuracy. Together, these improvements drive the accuracy and utility of the model in the dead sea bass detection task.

### 3.7. Experimental Analysis of Public Datasets

In order to validate the effectiveness of the YOLOv8n-Deadfish model proposed in this study for target detection in underwater images captured in real environments, the publicly available dataset Labeled Fishes in the Wild, provided by the National Marine Fisheries Service (NMFS) of the United States of America, was selected for evaluation in this paper. This dataset was constructed by Cutter et al. [40] and consists of images taken using a remotely operated vehicle (ROV) in a rocky reef area off southern California, USA, with precise bounding box annotations of fish targets. The images cover a wide range of fish, invertebrates, and typical seafloor environments, and are suitable for assessing tasks such as automated detection, identification, and tracking of underwater organisms. The dataset contains a total of 3167 images, which are divided into training, validation, and test sets in the ratio of 8:1:1 for model training and performance evaluation in this study. In the model training process, the Yolov8n model and YOLOv8n-Deadfish model were used for comparison.

The experimental results are shown in Table 4. Compared with the base model YOLOv8n, YOLOv8n-Deadfish improves the key metrics such as precision (P), recall (R), and mAP@0.5 by 1.1%, 1.0%, and 1.8%, respectively; at the same time, the number of parameters and the amount of floating-point operations are reduced by 23.3% and 18.5%, and the detection speed is improved from 336.1 FPS to 458.8 FPS, which shows better efficiency and lightweight advantages.

**Figure 13:**
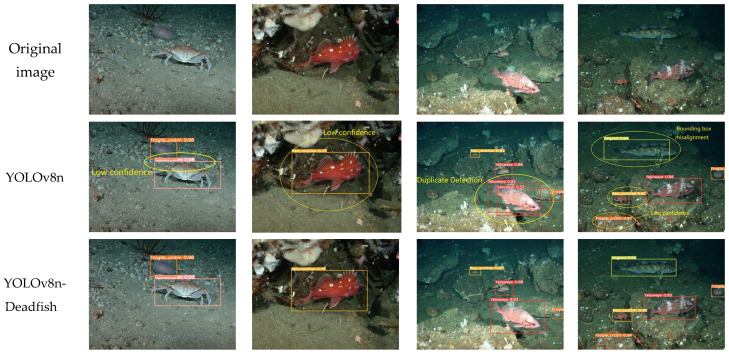
Detection effect for the public dataset Labeled Fishes in the Wild.

From the image detection results, as shown in Figure 13, YOLOv8n-Deadfish also shows stronger target perception and localization capabilities.

In the first column of the image, both models successfully recognize the target categories Fragile_urchin and Human_crab, but in contrast, YOLOv8n-Deadfish’s recognition confidence for Human_crab increases from 0.88 to 0.93, indicating that it has stronger discriminative ability in fine-grained target classification. In the second column of images, the recognition confidence of YOLOv8n on Red_rockfish is only 0.75, while the confidence of YOLOv8n-Deadfish is improved to 0.78, which further reflects that it is more accurate in recognizing target boundaries and more reliable in classifying targets in low-contrast complex backgrounds. In multi-target scenarios (e.g., the third column of the image), YOLOv8n misidentifies a Yelloweye fish as multiple overlapping targets with unstable detection frames, while YOLOv8n-Deadfish accurately identifies a single target with more compact and reasonable detection frames, and the identification is more stable. In the fourth column of the image, YOLOv8n’s detection frame for Lingcod is obviously small and inaccurate, while YOLOv8n-Deadfish’s detection frame is more accurately aligned and the target coverage is more comprehensive, which indicates that it has stronger feature extraction ability under the conditions of light change and background interference.

On the whole, YOLOv8n-Deadfish outperforms the original model in terms of bounding box fitting accuracy, target confidence, and small target recognition under occlusion, which effectively verifies the practicality and robustness of the structural optimization strategies proposed in this paper (e.g., the EMA attention mechanism and the BiFPN multi-scale feature fusion) in the complex underwater detection task.

### 3.8. Significance Analysis of Model Performance

To validate the stability and statistical reliability of the experimental results, five independent experiments were conducted on the dead sea bass dataset and a public dataset. In each experiment, only the random seed was changed to introduce randomness into the training process. The random seed is used to initialize the pseudo-random number generator (PRNG), where different seeds generate distinct pseudo-random sequences, leading to variations in the model training process (such as weight initialization, data shuffling, and data augmentation). Conversely, the same seed generates identical sequences, ensuring the reproducibility of the experiments.

#### 3.8.1. Statistical Significance Analysis of Model Performance Based on the Dead Sea Bass Dataset

Based on five independent experiments, this study applied statistical tests to analyze the performance differences among the models. Specifically, using YOLOv8n-Deadfish as the baseline model, we conducted a two-sample *t*-test to assess the significance of the experimental results of eight other mainstream detection models in terms of precision, recall, mAP@0.5, and inference speed (FPS), as shown in Table 5.

Among the multiple existing models compared with the YOLOv8n-Deadfish model, nearly all performance metrics (including recall, mAP@0.5, and detection speed) achieved a significance level of *p* = 0.000 (*p* < 0.01) in the *t*-test, indicating that the performance differences between these models and YOLOv8n-Deadfish in the aforementioned metrics are statistically significant. Although the difference in the precision metric between RT-DETRv2-R50 and YOLOv8n-Deadfish corresponds to a *p*-value of 0.009—which is slightly higher than that of the other comparisons—it is still less than 0.01, thus meeting the standard for statistical significance.

By integrating the results of multiple models under different random seeds and performing a *t*-test analysis, the YOLOv8n-Deadfish model demonstrated significant statistical advantages in accuracy, recall, detection precision, and inference speed on the dead sea bass dataset. This indicates that the performance improvements in the proposed YOLOv8n-Deadfish model in the dead sea bass detection task are not due to random fluctuations but represent substantial, stable, and statistically reliable enhancements.

#### 3.8.2. Significance Analysis of Model Performance on the Labeled Fishes in the Wild Public Dataset

To verify whether the performance improvement in the YOLOv8n-Deadfish model on the public dataset (Labeled Fishes in the Wild) is statistically significant, an independent-samples *t*-test was conducted on the results of YOLOv8n and YOLOv8n-Deadfish based on five independent experiments. The statistical results are presented in Table 6.

As shown in the table, the calculated t-values for the four key performance metrics—precision, recall, mAP@0.5, and detection speed—are all relatively high, and the corresponding *p*-values are all 0.000. This indicates that the performance differences between the two models on these metrics are statistically highly significant. The results further confirm that the performance improvement in YOLOv8n-Deadfish over the original YOLOv8n model on the public dataset, Labeled Fishes in the Wild, is not due to random fluctuations but rather reflects substantial improvements that are statistically validated.

## 4. Conclusions and Outlook

In this study, an improved YOLOv8n-Deadfish model is proposed to address the problems of high model complexity, insufficient accuracy of occluded target detection, and poor real-time performance in the task of dead sea bass detection in recirculating water aquaculture scenarios. In the dataset production stage, in order to improve the adaptive ability of the model in complex real-world environments, we introduced the environmental visual interference enhancement strategy (EDVA), which extends and enhances the original image data by simulating typical aquaculture interferences such as water turbidity, fish occlusion, and illumination changes so as to restore as realistically as possible the visual characteristics of dead sea bass in the actual aquaculture environment, and to provide the model training with more representative and challenging image samples for model training. On this basis, combined with systematic model optimization and innovation, the following main results were achieved:

First, in terms of feature extraction, a lightweight feature extraction module has been designed. By replacing the C2f module with the C2f-faster–EMA structure and combining it with partial convolution (PConv) and an efficient multi-scale attention mechanism (EMA), the number of network parameters and computational complexity have been effectively reduced. The number of parameters has been reduced from 3.0 M in the original model to 2.3 M, the floating-point operation amount is reduced from 8.1 G to 6.5 G, and the real-time detection speed is significantly improved, from 304.5 FPS to 385.2 FPS. Secondly, in terms of feature fusion, the weighted bidirectional feature pyramid network (BiFPN) is introduced. Through bidirectional cross-scale connections and feature weight allocation, the model achieves an efficient fusion of deep semantic information and shallow detailed features, improving the model’s detection accuracy for small, distant objects (overall mAP@0.5 improved by 1.5%); in addition, in terms of optimizing the performance of bounding box regression, the Inner-CIoU loss function is used instead of the traditional CIoU, which optimizes the convergence speed and generalization ability of bounding box regression, improving the model’s detection accuracy on the deadfish dataset by 1.0%. Finally, YOLOv8n-Deadfish achieved a detection speed of 424.6 FPS on the self-built dataset, an improvement of 39.4% over the original YOLOv8n. At the same time, the mAP@0.5 improved to 93.6%. The proposed YOLOv8n-Deadfish model outperforms mainstream classical detection models in key performance metrics, including precision (P), recall (R), mAP@0.5, and detection speed, enabling timely and efficient identification of dead sea bass. This study provides a methodological foundation and technical reference for the development of intelligent detection and monitoring technologies for dead sea bass in practical aquaculture environments.

In addition to its application in dead sea bass detection, the YOLOv8n-Deadfish model presented in this paper has the potential to be extended to other related tasks due to its lightweight structure and real-time detection capability. These tasks include underwater target monitoring, health assessments of other farmed species, and small-target detection scenarios in precision agriculture or environmental monitoring. Future research will further explore these application directions and assess the broad applicability of the model in resource-constrained environments.

Although typical disturbances in complex aquaculture environments, such as fish shading, water turbidity and light variations, were simulated as much as possible in the data collection and enhancement process in this study, it was still mainly conducted under semi-controlled conditions, which still represents a gap from the more complex and variable dynamic scenarios in real aquaculture systems. Therefore, the generalization ability and long-term stability of the model in real deployment environments still need to be further explored and verified.

In the subsequent research, it is necessary to further expand the data sources and collect image data across time and multiple environments in real aquaculture scenarios to improve the adaptability and robustness of the model; in addition, we will explore a lighter model structure or introduce the Transformer fusion architecture in the future in order to further improve the deployment efficiency and applicability of the model on edge devices. In addition, we will also try to introduce multimodal data such as water quality parameters and fish behavior to build a more comprehensive and intelligent aquatic health monitoring and early warning system, and promote the in-depth application of the model in real scenarios.

## Figures and Tables

**Table 1:** Training parameters.

Training Parameters	Numeric
Image size	640 × 640
Batch size	16
Learning rate	0.01
Iterations	300
Momentum	0.937
Weight decay	0.0005
Optimizer	SGD

**Table 2:** Results of ablation experiments.

Model	Precision/%	Recall/%	mAP@0.5/%	Number of Participants/M	Floating-Point Capacity/G	Detection Speed/FPS
YOLOv8n	88.0	89.0	92.3	3.0	8.1	304.5
YOLOv8n +C2f	86.4	86.8	90.3	2.2	6.3	394.2
YOLOv8n +C2f–EMA	87.5	88.3	91.1	2.3	6.5	385.2
YOLOv8n +C2f–EMA+BIFPN	89.4	88.8	92.6	2.3	6.5	384.5
YOLOv8n +C2f–EMA+BIFPN+ Inner-CIoU	90.0	90.4	93.6	2.3	6.6	424.6

**Table 3:** Performance comparison between different models.

Model	Precision/%	Recall/%	mAP@0.5/%	Number of Participants/M	Floating-Point Capacity/G	Detection Speed/FPS
Faster R-CNN	76.8	69.3	72.9	136.7	401.7	38.1
SSD	80.6	79.2	53.8	26.3	282.0	53.9
YOLOv5s	88.1	88.4	91.0	7.2	16.5	228.0
YOLOv7-tiny	86.2	87.1	91.8	6.0	13.1	252.0
YOLOv8n	88.0	89.0	92.3	3.0	8.1	304.5
YOLOv10n	87.4	88.0	91.0	2.7	8.4	274.9
YOLOv12n	88.1	88.4	91.6	2.6	6.5	288.8
RT-DETRv2-R50	89.5	88.8	92.7	36	100	114.5
YOLOv8n-Deadfish	90.0	90.4	93.6	2.3	6.6	424.6

**Table 4:** Experimental results of the public dataset Labeled Fishes in the Wild.

Model	Precision/%	Recall/%	mAP@0.5/%	Number of Participants/M	Floating-Point Capacity/G	Detection Speed/FPS
YOLOv8n	92.2	91.7	93.3	3.0	8.1	336.1
YOLOv8n-Deadfish	93.3	92.7	95.1	2.3	6.6	458.8

**Table 5:** Significance analysis of model performance of the dead sea bass dataset. Under this condition, √ denotes statistical significance (*p* < 0.01).

Metrics	Model	(x ± s)	t	*p*	Significance (*p* < 0.01)
Precision/%	Faster R-CNN	76.82 ± 0.26	93.055	0.000	√
	SSD	80.58 ± 0.24	70.063	0.000	√
	YOLOv5s	88.14 ± 0.34	10.695	0.000	√
	YOLOv7-tiny	86.22 ± 0.29	24.633	0.000	√
	YOLOv8n	88.00 ± 0.19	16.916	0.000	√
	YOLOv10n	87.44 ± 0.24	18.932	0.000	√
	YOLOv12n	88.10 ± 0.12	19.432	0.000	√
	RT-DETRv2-R50	89.48 ± 0.26	3.536	0.009	√
	YOLOv8n-Deadfish	89.98 ± 0.18	--	--	--
Recall/%	Faster R-CNN	69.26 ± 0.21	102.398	0.000	√
	SSD	79.18 ± 0.22	82.491	0.000	√
	YOLOv5s	88.36 ± 0.27	13.336	0.000	√
	YOLOv7-tiny	87.10 ± 0.22	24.262	0.000	√
	YOLOv8n	89.08 ± 0.19	10.422	0.000	√
	YOLOv10n	88.08 ± 0.19	18.318	0.000	√
	YOLOv12n	88.36 ± 0.21	15.361	0.000	√
	RT-DETRv2-R50	88.82 ± 0.26	10.571	0.000	√
	YOLOv8n-Deadfish	90.40 ± 0.21	--	--	--
mAP@0.5/%	Faster R-CNN	72.94 ± 0.34	118.496	0.000	√
	SSD	53.84 ± 0.19	330.708	0.000	√
	YOLOv5s	91.08 ± 0.29	16.124	0.000	√
	YOLOv7-tiny	91.78 ± 0.29	11.609	0.000	√
	YOLOv8n	92.30 ± 0.22	9.846	0.000	√
	YOLOv10n	91.02 ± 0.19	21.304	0.000	√
	YOLOv12n	91.56 ± 0.21	15.951	0.000	√
	RT-DETRv2-R50	92.70 ± 0.16	7.922	0.000	√
	YOLOv8n-Deadfish	93.58 ± 0.19	--	--	--
Detection speed/FPS	Faster R-CNN	38.12 ± 0.28	872.614	0.000	√
	SSD	53.88 ± 0.15	861.953	0.000	√
	YOLOv5s	227.96 ± 1.05	310.559	0.000	√
	YOLOv7-tiny	251.98 ± 1.01	278.408	0.000	√
	YOLOv8n	304.52 ± 1.25	171.049	0.000	√
	YOLOv10n	274.88 ± 0.76	275.218	0.000	√
	YOLOv12n	288.78 ± 1.14	204.689	0.000	√
	RT-DETRv2-R50	114.50 ± 1.37	415.948	0.000	√
	YOLOv8n-Deadfish	424.62 ± 0.95	--	--	--

**Table 6:** Significance analysis of model performance on the Labeled Fishes in the Wild dataset.Under this condition, √ denotes statistical significance (*p* < 0.01).

Metrics	Model	(x ± s)	t	*p*	Significance (*p* < 0.01)
Precision/%	YOLOv8n	92.18 ± 0.24	7.281	0.000	√
	YOLOv8n-Deadfish	93.26 ± 0.23			
Recall/%	YOLOv8n	91.68 ± 0.13	7.715	0.000	√
	YOLOv8n-Deadfish	92.68 ± 0.26			
mAP@0.5/%	YOLOv8n	93.32 ± 0.24	12.836	0.000	√
	YOLOv8n-Deadfish	95.08 ± 0.19			
Detection speed/FPS	YOLOv8n	336.10 ± 0.88	225.606	0.000	√
	YOLOv8n-Deadfish	458.76 ± 0.84			

## Data Availability

The data presented in this study are available upon request from the corresponding author. Due to project confidentiality, the dataset cannot be disclosed at this time.

## Abbreviations

The following abbreviations are used in this manuscript:

EMA	Efficient multi-scale attention
BiFPN	Bidirectional feature pyramid network
RAS	Recirculating aquaculture systems
CV	Computer vision
IoT	Internet of Things
CNN	Convolutional neural network
SSD	Single-Shot MultiBox Detector
RNN	Recurrent neural network
Inner-CIoU	Inner for complete intersection over union
CIoU	Complete intersection over union
PConv	Partial convolution
FLOPs	Floating-point operations
FPN	Feature pyramid network
PAN	Path Aggregation Network
mAP	Mean average precision

## References

[1] Garlock T., Asche F., Anderson J., Bjørndal T., Kumar G., Lorenzen K., Ropicki A., Smith M.D., Tveterås R. (2020). A global blue revolution: Aquaculture growth across regions, species, and countries. Rev. Fish. Sci. Aquac..

[2] Chen L., Yang X., Sun C., Wang Y., Xu D., Zhou C. (2020). Feed intake prediction model for group fish using the MEA-BP neural network in intensive aquaculture. Inf. Process. Agric..

[3] Gao Y., Zhang H., Peng C., Lin Z., Li D., Lee C.T., Wu W.-M., Li C. (2021). Enhancing nutrient recovery from fish sludge using a modified biological aerated filter with sponge media with extended filtration in aquaponics. J. Clean. Prod..

[4] Papadakis V.M., Glaropoulos A., Kentouri M. (2014). Sub-second analysis of fish behavior using a novel computer-vision system. Aquac. Eng..

[5] Saleh A., Sheaves M., Rahimi Azghadi M. (2022). Computer vision and deep learning for fish classification in underwater habitats: A survey. Fish Fish..

[6] Barbedo J.G.A. (2022). A review on the use of computer vision and artificial intelligence for fish recognition, monitoring, and management. Fishes.

[7] Monteiro F., Bexiga V., Chaves P., Godinho J., Henriques D., Melo-Pinto P., Nunes T., Piedade F., Pimenta N., Sustelo L. (2023). Classification of fish species using multispectral data from a low-cost camera and machine learning. Remote Sens..

[8] Yang X., Zhang S., Liu J., Gao Q., Dong S., Zhou C. (2021). Deep learning for smart fish farming: Applications, opportunities and challenges. Rev. Aquac..

[9] Zhou C., Wang C., Sun D., Hu J., Ye H. (2025). An automated lightweight approach for detecting dead fish in a recirculating aquaculture system. Aquaculture.

[10] Hasan N., Ibrahim S., Aqilah Azlan A. (2022). Fish diseases detection using convolutional neural network (CNN). Int. J. Nonlinear Anal. Appl..

[11] Zhao Z., Liu Y., Sun X., Wang L., Zhang M. (2021). Composited FishNet: Fish Detection and Species Recognition from Low-Quality Underwater Videos. IEEE Trans. Image Process..

[12] Li Y., Zhu D., Fan H.D. (2021). An Improved Faster R-CNN Marine Fish Classification Identification Algorithm. Proceedings of the 2021 2nd International Conference on Artificial Intelligence and Computer Engineering (ICAICE).

[13] He K., Gkioxari G., Dollár P., Girshick R. Mask R-CNN. Proceedings of the IEEE International Conference on Computer Vision.

[14] Jiang P., Ergu D., Liu F., Cai Y., Ma B. (2022). A Review of YOLO Algorithm Developments. Procedia Comput. Sci..

[15] Liu W., Anguelov D., Erhan D., Szegedy C., Reed S., Fu C.Y., Berg A.C. (2016). SSD: Single Shot Multibox Detector. Computer Vision—ECCV 2016, Proceedings of the 14th European Conference, Amsterdam, The Netherlands, 11–14 October 2016, Part I.

[16] Zhao J., Bao W., Zhang F., Zhang L., Zhang W. (2018). Modified Motion Influence Map and Recurrent Neural Network-Based Monitoring of the Local Unusual Behaviors for Fish School in Intensive Aquaculture. Aquaculture.

[17] Jäger J., Rodner E., Denzler J., Schöning J., Dengel A. SeaCLEF 2016: Object Proposal Classification for Fish Detection in Underwater Videos. Proceedings of the CLEF (Working Notes).

[18] Li J., Xu C., Jiang L., Pan Y., Chen H. (2019). Detection and Analysis of Behavior Trajectory for Sea Cucumbers Based on Deep Learning. IEEE Access.

[19] Yang S.P., Li H., Liu J.J., Zhang Y., Wang X. (2024). A Water Surface Dead Fish Detection Method Based on Multi-Scale Feature Fusion and Attention Mechanism. J. Zhengzhou Univ. (Nat. Sci. Ed.).

[20] Cai K., Miao X., Wang W., Wang J. (2020). A Modified YOLOv3 Model for Fish Detection Based on MobileNetv1 as Backbone. Aquac. Eng..

[21] Li S., Li C., Yang Y., Zhang Q., Wang Y., Guo Z. (2022). Underwater scallop recognition algorithm using improved YOLOv5. Aquac. Eng..

[22] Zhao S., Zhang S., Lu J., Zhang C. (2022). A Lightweight Dead Fish Detection Method Based on Deformable Convolution and YOLOv4. Comput. Electron. Agric..

[23] Zhang J., Li X., Wang Y., Liu H. (2024). A Method for Counting Fish Based on Improved YOLOv8. Aquac. Eng..

[24] Chen N., Zhu J., Zheng L. (2024). Light-YOLO: A Study of a Lightweight YOLOv8n-Based Method for Underwater Fishing Net Detection. Appl. Sci..

[25] Zhang Z., Wang M., Li Y., Huang J. (2024). An Improved YOLOv8n Used for Fish Detection in Natural Water Environments. Animals.

[26] Shah C., Kumar A., Rana M., Singh P., Li J. (2025). Yolov8-tf: Transformer-Enhanced YOLOv8 for Underwater Fish Species Recognition with Class Imbalance Handling. Sensors.

[27] Terven J., Córdova-Esparza D.M., Romero-González J.A. (2023). A Comprehensive Review of YOLO Architectures in Computer Vision: From YOLOv1 to YOLOv8 and YOLO-NAS. Mach. Learn. Knowl. Extr..

[28] Chen J., Kao S., He H., Sun Y., Zhang X., Xu W. Run, Don’t Walk: Chasing Higher FLOPS for Faster Neural Networks. Proceedings of the IEEE/CVF Conference on Computer Vision and Pattern Recognition (CVPR).

[29] Liu X., Peng H., Zheng N., Wang M., Yu S., Zhan X., Lin D. EfficientViT: Memory Efficient Vision Transformer with Cascaded Group Attention. Proceedings of the IEEE/CVF Conference on Computer Vision and Pattern Recognition (CVPR).

[30] Woo S., Park J., Lee J.Y., Kweon I.S. CBAM: Convolutional Block Attention Module. Proceedings of the European Conference on Computer Vision (ECCV).

[31] Hu J., Shen L., Sun G. Squeeze-and-Excitation Networks. Proceedings of the IEEE Conference on Computer Vision and Pattern Recognition (CVPR).

[32] Li X., Zhong Z., Wu J., Yang Y. Expectation-Maximization Attention Networks for Semantic Segmentation. Proceedings of the IEEE/CVF International Conference on Computer Vision (ICCV).

[33] Zheng Z., Wang P., Liu W., Li J., Ye R., Ren D. Distance-IoU Loss: Faster and Better Learning for Bounding Box Regression. Proceedings of the AAAI Conference on Artificial Intelligence.

[34] Tong Z., Wang Y., He Z., Yu H., Zhang X. (2023). Wise-IoU: Bounding Box Regression Loss with Dynamic Focusing Mechanism. arXiv.

[35] Zhang H., Xu C., Zhang S. (2023). Inner-IoU: More Effective Intersection over Union Loss with Auxiliary Bounding Box. arXiv.

[36] Lv W., Zhang Y., Liu Q., Huang G., Li S. (2024). RT-DETRv2: Improved Baseline with Bag-of-Freebies for Real-Time Detection Transformer. arXiv.

[37] Wang C.Y., Bochkovskiy A., Liao H.Y.M. YOLOv7: Trainable Bag-of-Freebies Sets New State-of-the-Art for Real-Time Object Detectors. Proceedings of the IEEE/CVF Conference on Computer Vision and Pattern Recognition (CVPR).

[38] Guan S., Zhang Y., Liu M., Chen J., Li Q. (2024). Real-Time Detection and Counting of Wheat Spikes Based on Improved YOLOv10. Agronomy.

[39] Sapkota R., Karkee M. (2025). Improved YOLOv12 with LLM-Generated Synthetic Data for Enhanced Apple Detection and Benchmarking against YOLOv11 and YOLOv10. arXiv.

[40] Cutter G., Stierhoff K., Zeng J. (2015). Automated Detection of Rockfish in Unconstrained Underwater Videos Using Haar Cascades and a New Image Dataset: Labeled Fishes in the Wild. Proceedings of the 2015 IEEE Winter Applications and Computer Vision Workshops (WACVW).

